# Molecular Mechanisms of DNA Replication Checkpoint Activation

**DOI:** 10.3390/genes5010147

**Published:** 2014-03-06

**Authors:** Bénédicte Recolin, Siem van der Laan, Nikolay Tsanov, Domenico Maiorano

**Affiliations:** 1 Institute of Human Genetics, CNRS-UPR1142, Department “Molecular Bases of Human Diseases”, 141, Rue de la Cardonille, Montpellier 34396 Cedex 5, France; E-Mails: benedicterecolin@hotmail.com (B.R.); siem.vanderlaan@igh.cnrs.fr (S.L.); 2 RNA Biogenesis Laboratory, Institute of Molecular Genetics of Montpellier, 1919 Route de Mende, Montpellier 34293, France; E-Mail: nikolay.tsanov@igmm.cnrs.fr

**Keywords:** ATR, DNA replication fork arrest, DNA replication stress, DNA damage, single**-**stranded DNA

## Abstract

The major challenge of the cell cycle is to deliver an intact, and fully duplicated, genetic material to the daughter cells. To this end, progression of DNA synthesis is monitored by a feedback mechanism known as replication checkpoint that is untimely linked to DNA replication. This signaling pathway ensures coordination of DNA synthesis with cell cycle progression. Failure to activate this checkpoint in response to perturbation of DNA synthesis (replication stress) results in forced cell division leading to chromosome fragmentation, aneuploidy, and genomic instability. In this review, we will describe current knowledge of the molecular determinants of the DNA replication checkpoint in eukaryotic cells and discuss a model of activation of this signaling pathway crucial for maintenance of genomic stability.

## 1. Introduction

Perpetuation of life requires duplication of the genetic information stored in the DNA of each cell of a given organism. In all kingdoms of life, amazingly efficient replication machineries achieve the duplication of the genome. In eukaryotes, DNA synthesis is a challenging task since the replication machinery has to deal with many obstacles, such as specialized DNA structures, chromatin, and sometimes with damaged DNA. Nevertheless, the duplication process must be as faithful as possible to minimize replication errors that can lead to mutagenesis. Pioneering studies of eukaryotic chromosome replication using *in vitro* extracts derived from mammalian cells infected with the SV40 virus have revealed similar principles to prokaryotic replication. However, the replication of the cellular genome turned out to be an incredibly complex process involving (so far known) as many as 60 different proteins, the function of which is still not completely understood. Probably the main reason that can explain this difference is that DNA replication is tightly linked to progression of the cell cycle and unlike other cellular processes, such as transcription and translation, replication only occurs once, and only once in each cell cycle. In the following sections, we will introduce some general basic principles of DNA replication. More in-depth information can be found in recent reviews published on this subject [1,2,3].

## 2. Principles of Eukaryotic DNA Replication

### 2.1. Pre-RC Formation

**Figure 1:**
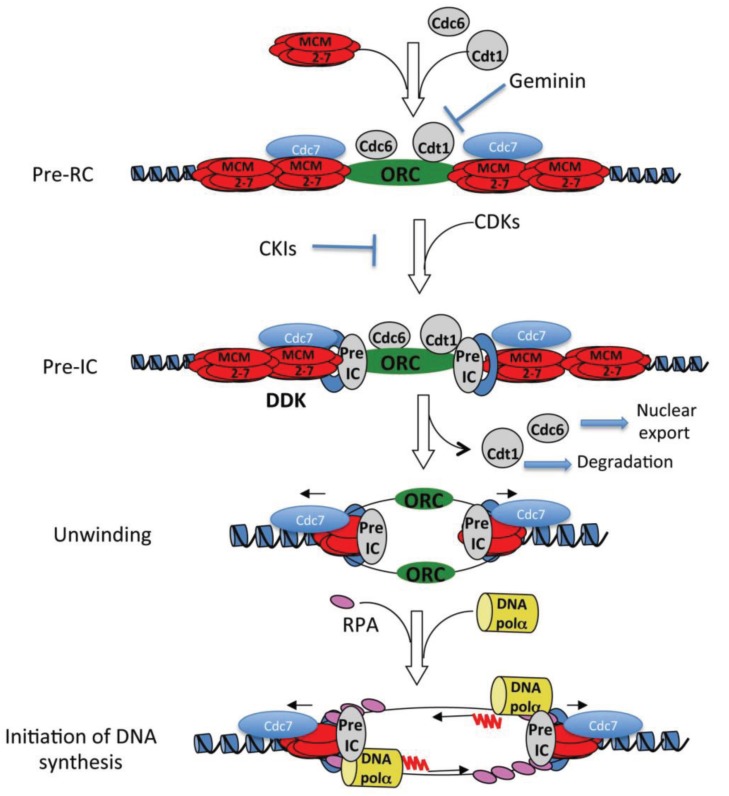
Schematic model of initiation of DNA synthesis. For sake of clarity, a number of proteins that make the pre-initiation complex (pre-IC) have been grouped in one complex. These include Cdc45, the GINS complex, MCM10, TopBP1, SLD2 and SLD3. A ring-like complex of proteins (in blue in the figure) made of Claspin, Timeless and Tipin make the Fork Protection Complex that tethers the GMC helicase to the DNA polymerases. RNA primers (red waves) are made by the catalytic activity of DNA primase and elongated by the catalytic activity of DNA polymerase α (black arrows linked to red waves).

Replication of the DNA requires opening of the double helix to separate the two individual DNA strands, a process called melting or unwinding. This process is essential to allow DNA polymerases (the enzymes that catalyze the DNA synthesis reaction) to copy the genetic information by synthesis of new DNA chains on both DNA strands. Unwinding occurs at specific sites along the chromosomes, the so-called DNA replication origins. Unlike prokaryotes in which unwinding is essentially catalyzed by one protein (DnaA), in eukaryotes this process requires a sophisticated multi-protein complex including the Origin Recognition Complex (ORC) that binds to DNA replication origins ([4] for review), and two additional proteins, CDC6 and CDT1 (Figure 1). This multi-protein complex, allows recruitment of the putative replicative helicase, the MCM2-7 protein complex, in an ATP-dependent manner. This reaction is currently known as “*licensing*” or pre-RC formation. Structural *in vitro* studies of the budding yeast MCM2-7 complex from reconstituted pre-RC revealed that MCM2-7 is loaded on DNA as a salt-resistant head-to-head double hexamer in an ATP-dependent reaction [5,6]. This configuration is thought to be necessary for initiation of a pair of bidirectional replication forks. More recently, a salt-sensitive intermediate of this reaction that contains a single ORC, CDC6, and MCM2-7 (OCM complex) was identified [7]. Hence, it was proposed that in budding yeast CDT1 recruits MCM2-7 to an ORC-CDC6 complex on DNA in two subsequent rounds prior to formation of the MCM2-7 double hexamer. Hydrolysis of ATP by ORC1 and CDC6 is required for both rounds, and can also lead to MCM2-7 release if components of the pre-RC are missing [7,8]. In metazoans, the Geminin protein, an inhibitor of DNA replication [9], blocks pre-RC formation by interfering with the activity of chromatin-bound CDT1 ([10,11] and Figure 1). MCM9, an MCM2-7-related protein that appeared during evolution in multicellular organisms [12], has also been implicated in pre-RC formation in *Xenopus* [13], although its role in this reaction is not currently clear [14]. Instead, recent data clearly show an important function for this protein, in a complex with another multicellular-specific MCM2-7-related protein, MCM8, in homologous recombination [15,16,17]. The MCM2-7 complex is curiously recruited in multiple copies adjacent to DNA replication origins, but it is currently unclear whether upon recruitment, an initial, even partial melting of the double helix occurs. Notwithstanding, full unwinding activity of the MCM2-7 helicase depends upon additional factors, such as the CDC7 protein kinase, Dpb11/Cut5/Rad4/TopBP1, CDC45, the GINS complex and MCM10 (this latter being unrelated to the MCM2-7 protein family) themselves recruited onto pre-RCs ([18] for review).

### 2.2. Pre-IC Formation

Loading of additional factors onto the pre-RC, namely the CDC45 protein and the GINS complex, marks formation of the pre-Initiation Complex (pre-IC) whose assembly requires Cyclin-Dependent Kinase (CDK) activity. Hence, this reaction can be specifically inhibited by CDK Inhibitors (CKI), such as p21 or p27 (Figure 1). CDK activity is believed to be important to phosphorylate key factors of pre-IC to activate them (see below). CDC45, in turn, allows assembly of initiation complexes by recruitment of DNA polymerases α and δ at replication origins [19,20,21]. Recent studies have shown that the MCM2-7 complex physically interacts with CDC45 and the GINS complex forming the so-called CMG complex ([22] for review). This complex is believed to constitute the active form of the replicative helicase. Structural studies have shown that the CDC45 protein binds to the MCM2-7 complex onto chromatin and stabilizes the ring-shaped structure of this helicase onto the DNA [23]. The CDC7 protein kinase, in a complex with its regulatory subunits Dbf4 or Drf1 (also called DDK, for Dbf4- or Drf1-Dependent Kinase), phosphorylates MCM2-7 subunits and stimulates MCM2-7 function probably facilitating a conformational change in the helicase complex [24,25]. In yeast, two additional proteins, SLD2 and SLD3, have also been shown to be required at this step of initiation of DNA synthesis. Their phosphorylation by CDKs is an essential step in formation of pre-ICs [26,27] and is believed to regulate formation of a complex between Dpb11/TopBP1, SLD2, GINS and DNA polymerase ε, and its subsequent recruitment on DNA [26,27,28,29]. Another protein, SLD7, was shown to form a complex with SLD3 and CDC45, which associates with early-firing origins in a DDK-dependent manner and might regulate timing of origin firing in yeast [30,31]. In the future, it will be interesting to determine the structural architecture of this multi-protein complex. 

### 2.3. The Initiation Complex (IC)

Once activated, the helicase unwinds the DNA thus generating long stretches of single-stranded DNA (ssDNA). This reaction introduces topological stress downstream of the unwound DNA, because intact DNA molecules are covalently closed. This would not be the case if the DNA is cleaved on one strand for instance, since the cleaved DNA strand could freely rotate around the unbroken strand and relieve the topological stress introduced by the helicase. Topological stress is removed by the action of Topoisomerases, enzymes that catalyze the cleavage and reseal of one (type I and III topoisomerases) or two (type II topoisomerases) DNA strands. Although *in vitro* ssDNA is a substrate for DNA polymerases, the recruitment of the latter *in vivo* depends on the previous binding of the heterotrimeric RPA complex, the major ssDNA binding protein. RPA binding stimulates DNA polymerase activity onto ssDNA [21,32] and binding of RPA itself may be mediated by the helicase during the unwinding reaction [33]. RPA-loaded ssDNA is now competent to recruit the DNA polymerase α/primase holoenzyme (Figure 1) which is made of five distinct polypeptides. This complex has the unique property to initiate DNA synthesis *de novo*, that is to say, in the absence of a 3' hydroxyl (OH) end. This is due to its associated primase activity which synthesizes short, 10 bases-long, ribonucleotides stretches at melted replication origins, that is extended by the catalytic subunit of DNA polymerase α (p180). These replication intermediates are then the substrate of another DNA polymerase, Pol δ, whose activity is much more processive than that of Pol α and allows efficient replication of the whole genome. This is mainly due to complex formation with PCNA, a replication factor that functioning as DNA polymerase δ cofactor, strongly stimulates its polymerase activity. PCNA forms a homotrimeric ring and is loaded around the DNA in a reaction catalyzed by RF-C. This pentameric protein complex catalyzes opening of the PCNA ring in an ATP-dependent manner and resealing it around the DNA [34]. In this reaction, RF-C recognizes the 3' hydroxyl (OH) ends generated by the action of DNA polymerase (see Bloom’s chapter in the same special issue). Given the asymmetry of the DNA molecule, DNA synthesis must occur continuously on one strand (leading) and discontinuously on the opposite strand (lagging strand). Genetic studies in yeast have suggested that another polymerase, DNA polymerase ε, which is also required for chromosomal DNA replication, functions on the leading strand DNA synthesis [35], and is required for DNA replication in *Xenopus* [36]. Hence, current models of eukaryotic DNA replication propose that efficient, bi-directional DNA synthesis occurs through the participation of three replicative DNA polymerases. The structural basis of this complex is still not well understood, although an interesting model has been proposed in [37].

### 2.4. Elongation and Termination

Once the three replicative DNA polymerases are loaded onto the DNA, the replisome is now formed. The MCM2-7-related helicase MCM8 was shown to be incorporated to the replisome at this stage [38,39,40], contributing to fork progression probably through specialized DNA or chromatin structures and facilitating replication fork restart by homologous recombination [15]. The replication machinery is able to duplicate the genome at the amazing average speed of 3000 bp per minute [41], making very few or no mistakes. Additional factors are required in the maturation and sealing of DNA chains on the lagging strand. Fen1 exonuclease removes the RNA primers from the Okazaki fragments, and DNA ligase I seals the gaps left between Okazaki fragments [34]. The above-described proteins constitute the basic components of the replication machinery, however other components are also present *in vivo* facilitating replication through regions of the genome difficult to replicate, including several DNA helicases, such as Werner and Bloom helicases, as well as translesion DNA polymerases [42]. DNA synthesis is bidirectional, that is to say two replisomes originating from one single replication origin polymerize DNA chains spreading outwards from the origin, making the basic element of a replication domain, also called the replicon [43]. Two replisomes will encounter each other when two replicons have been completely replicated. At this stage, the replisome has to be unloaded from chromatin. The mechanisms responsible for unloading of the replisome are to date not well understood. One possibility might be that collision between two replisomes induces formation of a structure resembling a stalled fork that activates accessory proteins facilitating replisome unloading. Recently, it has been reported that the MCM-BP protein, may function as unloader of the MCM2-7 complex form replicated chromatin, although the molecular mechanism of this reaction remains unknown [44].

## 3. Cell Cycle Regulation of DNA Synthesis

### 3.1. The Licensing Factor Hypothesis

Unlike other cellular processes, such as transcription and translation, DNA synthesis occurs once, and only once during one cell cycle. This stringent control of DNA replication ensures that no regions of the DNA will be replicated more than once, thus preventing gene amplification that could be deleterious for the cell, as it is known to be the case in cancer cells. Pioneering experiments of mammalian cell fusions have shown that nuclei in the G2-phase of the cell cycle are refractory to rereplication and suggested the presence of a diffusible factor that represses re-initiation of DNA synthesis within S phase [45]. Further studies using *in vitro Xenopus* egg extracts have shown that permeabilization of the nuclear membrane of G2 nuclei is sufficient to induce re-replication, and led to the proposal of Licensing Factor (LF) as a diffusible factor that restrains S-phase to only once-per-cell cycle. LF is essential for initiation of DNA synthesis and is recruited onto chromatin following nuclear envelope breakdown in M-phase [46], binding to chromatin prior initiation of DNA synthesis to give the license to replicate. Currently the “*licensing*” term is referred to as the loading of the MCM2-7 helicase to replication origins [47,48,49,50]. MCM2-7 proteins are removed from chromatin during replication, and they cannot rebind because LF is inactivated (either degraded or exported from the nucleus, see below). Cells reacquire the license to replicate only after passing through mitosis, when LF becomes reactivated. Biochemical studies in *Xenopus* [10,51] have led to the identification of the CDT1 protein (see Section 2.1) as the best candidate for LF. Evidence for a similar mechanism has been provided in mammalian cells [11,52]. CDT1 activity is under tight control; on one hand, CDT1 activity is repressed by Geminin in S-phase and M-phase, and on the other hand, CDT1 is degraded upon initiation of DNA synthesis [53], by a PCNA-dependent mechanism [54]. Consistent with this tight regulation, alteration of CDT1 abundance causes re-replication [55,56,57,58] and interestingly, the extent of re-replication is increased when checkpoints are inhibited [59,60], showing that the checkpoint is also able to monitor the extent of DNA synthesis when the licensing control is disrupted. To date, the DNA structures that activate the replication checkpoint upon re-replication are not known. In yeast, there is no nuclear membrane breakdown in M-phase; hence, licensing regulation is somehow different. In budding yeast, CDT1 enters the nucleus together with the MCM2-7 complex and is then exported to the cytoplasm after initiation of DNA synthesis [61]. Curiously, in fission yeast CDT1 is degraded as in vertebrates after initiation of DNA synthesis, however re-replication requires co-expression with CDC6 [62].

### 3.2. Regulation of DNA Synthesis by CDKs

As for other cell cycle transitions, S-phase progression depends on the activity of CDKs, specific threonine-serine protein kinases that phosphorylate specific substrates leading to their activation or inactivation at specific cell cycle stages. CDKs activity is in part regulated by cell cycle-dependent association with a regulatory subunit, a Cyclin, whose expression and/or stability is cell cycle-dependent. Association between a CDK and a specific Cyclin controls cell cycle transitions. For instance, CDK2, in a complex with Cyclin E, is believed to be important in controlling the G1 to S phase transition, while the CDK2/Cyclin A complex is important for S phase progression. In addition, the CDK/Cyclin complex is kept in an inactive form by phosphorylation of specific residues within this complex. This inhibitory phosphorylation is then removed by protein phosphatases of the CDC25 family (A through C) resulting in activation of the CDK kinase [63]. CDC25A dephosphorylates two inhibitory phosphorylations on residues threonine 14 and tyrosine 15 located within the ATP binding loop on CDK1 and CDK2. CDK activity is also regulated by specific inhibitors, the CDK inhibitors or CKI [64]. Interestingly, analysis of gene function by targeted disruption in mouse has shown that several CDK, such as CDK2 for instance, is not essential for embryonic development [65]. This is probably due to functional redundancy within the CDK family, and a previous study in yeast has indeed shown that only one CDK1/Cyclin B1 complex is sufficient to drive a full cell cycle [66].

### 3.3. G1 to S-Phase Transition

In mammalian cells, the activity of CDKs, mainly CDK2/Cyclin E and that of CDK4/Cyclin D1, has been shown to be important for G1 to S-phase transition. While CDK4/Cyclin D1 appears to be important in activating the S-phase specific transcriptional program that derepresses E2F-specifc genes through phosphorylation of the retinoblastoma protein, the CDK2/CyclinE complex seems to be important in activating the pre-IC complex. To date, the key substrates phosphorylated by this kinase are not well known, but the SLD2 and SLD3 proteins appear to be good candidates [26,27]. Their phosphorylation could facilitate protein-protein interactions within the pre-IC (namely by creating a docking site for TopBP1/Dpb11) thus facilitating MCM2-7 helicase activation. Vertebrate orthologous of these genes have not yet been clearly identified. Ticrr/Treslin [67,68] and GEMC1 [69] have been proposed as putative candidate for SLD3 orthologues although they do not appear to complement mutation in the corresponding yeast mutants [70]. The RecQL4 helicase has been proposed as SLD2 orthologous, although the vertebrate protein is much bigger than the yeast counterparts. Importantly, low CDK activity is required to assemble pre-RC, and a premature raise of CDK activation inhibits origin licensing, while inhibition of CDK activity in G2 facilitates re-replication [71]. The combined action of DDK and CDK activity is required for S phase progression by phosphorylating specific targets [28]. On one hand, CDK2/Cyclin A targets pre-RC components to inactivate them, while on the other hand, it targets components of the replication forks, such as RF-C and DNA ligase, stimulating their activity [72].

## 4. DNA Damage Response during S-Phase

Throughout S-phase, replisomes can encounter physiological or environmentally created obstacles that disturb their stability and partially block their progression. In this case, replication fork is said to have “stalled”. This replication stress event inhibits origin firing in budding yeast and mammalian cells through the activation of DNA Damage Response (DDR) pathway [73]. In this section, we will present the different DNA structures that can promote the DDR during the S-phase and the molecular bases of this pathway.

### 4.1. Sources of DNA Damage Acting as Obstacle for the Replisome

During S-phase, replicating DNA is particularly sensitive to DNA-damaging assaults. DNA breaks are the most common lesions experienced by the genome. The study of DNA breaks has received considerable attention since they are generated during physiological processes such as homologous recombination in meiosis, maturation of B and T lymphocytes or by the action of the enzymes involved in DNA compaction, such as DNA topoisomerases [74]. In addition, DNA-damaging agents can directly or indirectly induce DNA breaks, which are a major source of chromosome rearrangement and genome instability. The presence of breaks in S-phase can lead to replication forks collapse followed by recombination resulting in genetic rearrangements [75]. On the Earth’s surface, the most powerful environmental DNA-damaging source remains the ultra-violet (UV) light unabsorbed by the ozone layer and which modifies DNA bases pairing. Within the solar spectrum, UV-A light (320–400 nm) induces Reactive Oxygen Species (ROS) formation that generates DNA damage in cells, and this is the reason why this part of UV light is considered like the most genotoxic wavelength. UV-B (280–320 nm) and UV-C (100–280 nm) can be directly absorbed by the DNA molecule producing covalent links between two adjacent pyrimidine bases and leads to formation of cyclobutane pyrimidine dimers and 6-4 photoproducts. These structural modifications induce the distortion of the DNA double helix preventing the progression of the regular replication machinery [76,77]. Since the DNA replication fork is asymmetric, it is generally accepted that the location of DNA lesions on the replication fork templates can induce different cellular responses. Lagging-strand template lesions constitute few obstacles to replication fork progression since DNA synthesis is already discontinuous and would leave a gap with a 5' primer end. In contrast, leading-strand template damage induces a more pronounced arrest of DNA synthesis and requires repriming to resume DNA synthesis.

### 4.2. The Molecular Bases of the DNA Damage Response during S-Phase

**Figure 2:**
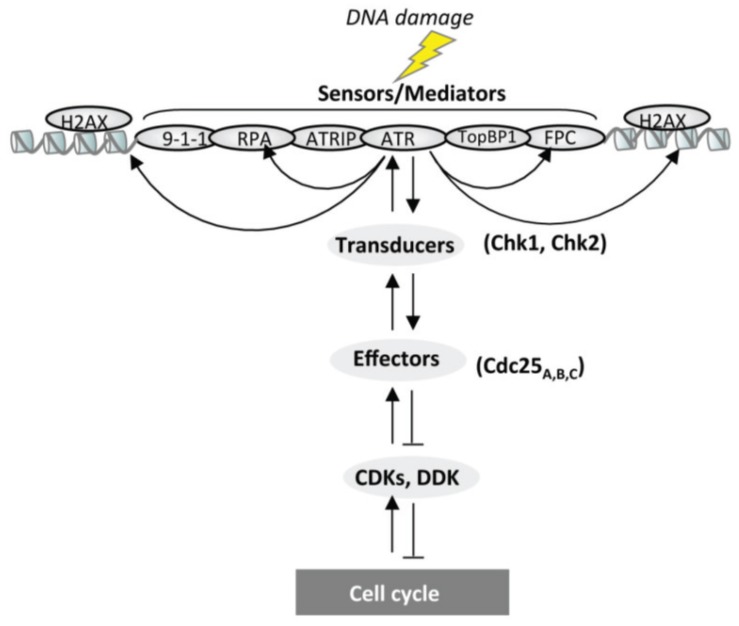
Schematic representation of the replication checkpoint pathway activated following DNA damage. DNA damage generated onto the DNA induces activation and/or relocalization of sensor and mediator proteins triggering a phosphorylation cascade (downwards arrows) that in turn activates soluble, as well as chromatin-bound, transducers and effector proteins resulting in cell cycle delay. Once the damage has been repaired signaling is inactivated by dephosphorylation (backwards arrows). FPC stands for Fork Protection Complex and includes Timeless, Claspin and Tipin proteins.

DDR is activated in response to DNA injuries. Depending on the type and the extent of damage, DDR can drive cells into apoptosis or senescence, or allows cell survival by activating specific mechanisms such as DNA repair or DNA damage tolerance. The proteins involved in these signaling pathways (Figure 2 and Table 1) are hierarchically classified into three categories: sensor and mediator proteins that recognize the lesion and generate the activation signal; transducer proteins that relay and amplify the initiated signal, and finally effector proteins that connect the signal activation with the cell cycle machinery [78]. The signal transduction pathways of the DDR that promotes cell cycle arrest during ongoing DNA synthesis will be designed as DNA Replication Checkpoint.

At the apex of the DDR are three kinases belonging to the phosphatidylinositol-3-OH kinase (PI3K or PIKK) family: ATM (Ataxia Telangiectasia Mutated), ATR (ATM-and Rad3-related) and DNA-PK (DNA-dependent protein kinase). In addition, the Poly (ADP-Ribose) Polymerase-1 (PARP-1) enzyme also appears to play a key role at the earliest step of DNA damage signaling ([78] for review). DNA double-strand breaks activate PARP-1, ATM and DNA-PK, while ATR is mostly involved in monitoring DNA replication stress and other types of DNA damage. Once activated, these kinases phosphorylate a conserved motif S/TQ on their substrates. A genome-wide approach has identified as many as seven hundred potential substrates of these kinases [79]. It is interesting to note that until very recently, ATR, and not ATM, has been considered to be an essential gene for viability in human and murine cells [80,81]. However, more recent data now show that also ATM is required for embryonic development [82,83]. The ATR-dependent replication checkpoint pathway constitutes a crucial barrier required to prevent propagation of mutated-DNA. Hence, identifying which specific structures activate ATR, and characterizing in-depth all the mechanisms underlying this specific signaling pathway, appears like a key step in understanding maintenance of genetic stability. In the rest of this review, we will focus on the role and function of ATR activation at stalled replication forks, which constitutes the core of the replication checkpoint.

**Table 1:** List of proteins implicated in replication checkpoint activation from yeast to human. Question marks indicate possible homologous genes or those not yet identified homologous.

Species Gene name	*H. sapiens*	*X. laevis*	*D. melanogaster*	*C. elegans*	*Yeast* *(S. cerevisiae/S. pombe)*
ATR	ATR	ATR	Mei-41	atl-1	Mec1 S.c/Rad3 S.p.
ATRIP	ATRIP	ATRIP	mus 304	ATRIP	Ddc2/ATRIP
TopBP1	TopBP1	TopBP1	mus 101	mus 101	Dbp11/Cut5
Rad9	Rad9	Rad9	Rad9	Hrp-9	Ddc1/Rad9
Rad1	Rad1	Rad1	Rad1/Dmel	mrt-2	Rad17
Hus1	Hus1	Hus1	hus1	hus-1	Mec3/Hus1
Rad17	Rad17	Rad17	Rad17	hrp-17	Rad24/Rad17
Claspin	Claspin	Claspin	Claspin	F25H5.5	Mrc1/Mrc1
Timeless	Timl	Tim 1	Tim	tim-1	Tof1/Swi1
Tipin	Tipin	Tipin	Timeout	?	Csm3/Swi3
CHK1	CHK1	CHK1	grp1/DChk1	CHK1	Chk1/Rad27
CDC25A	CDC25A	CDC25A	String	CDC-25	CDC25

## 5. The Replication Checkpoint

### 5.1. The S-Phase and Intra S-Phase Checkpoint

Progression of DNA synthesis is continuously monitored by these sensing pathways that ensure both coordination of S-phase with other cell cycle phases as well as control over firing of additional replication origins. The function of the S-phase checkpoint is to delay the cell cycle, such as G1/S transition and G2/M through down regulation of CDK activity, mainly CDK2 and/or CDK1. The function of the intra-S phase checkpoint is to slow down DNA synthesis and to inhibit additional origin firing (mainly late origin firing), to facilitate DNA repair and as such avoid duplication of DNA discontinuities. Probably, the best example of activation of the replication checkpoint is when cells experience DNA damage, or nucleotides starvation, when entering S-phase. Since the G1 to S-phase transition relies upon the activation of CDKs and DDKs kinases, activation of the replication checkpoint results in inhibition of these two kinases. In yeast, the checkpoint kinase Rad53 has been shown to phosphorylate DBF4 and SLD3 and by consequence to inhibit further origin firing [84]. Inactivation of CDK2 is well described and relies upon rapid proteasomal degradation of CDC25A [85], a phosphatase that removes an inhibitory phosphorylation on CDK2 (see Section 3.2). In the presence of very low levels of CDC25A, CDK2/Cyclin E remains in an inactive form thus blocking S-phase entry and/or activation of additional origins. ATM and ATR can also phosphorylate DDKs, in particular CDC7/DBF4. However, the effect of checkpoint activation on the kinase activity of this complex is controversial. Previous work has shown that activation of the intra S-phase checkpoint inactivates CDC7, by dissociation of DBF4 from chromatin [86]. This conclusion has been recently challenged [87] and very recent results in both budding yeast and mammalian cells suggest that ATM/ATR-dependent phosphorylation of CDC7 does not affect its kinase activity, which is important to inhibit rereplication within S-phase [88]. In sum, these results suggest that, while in the absence of damage, phosphorylation of CDC7/Dbf4 by CDKs is important for origin activation [28], ATM/ATR-dependent phosphorylation inhibits DNA synthesis and/or origin firing during the DNA damage response although the kinase activity of CDC7/DBF4 appears to be essential to prevent replication fork collapse.

### 5.2. Generation of ssDNA after DNA Damage or Replicative Stress

**Figure 3:**
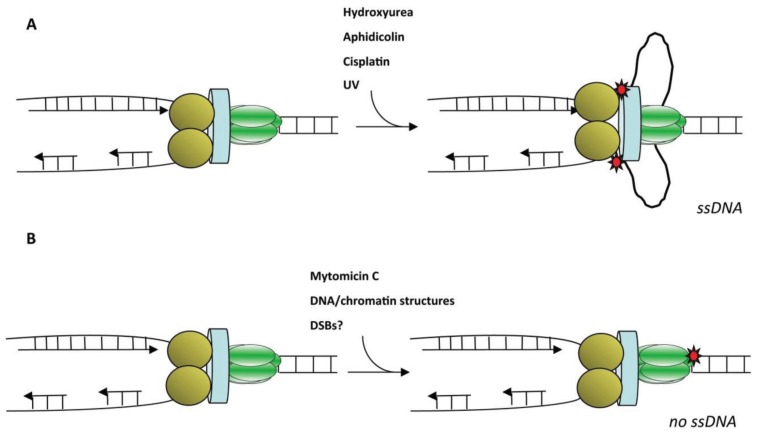
Schematic representation of two different types of replication stress. (**A**) Progression of a replication fork is represented by leading and lagging strand DNA polymerases (yellow circles) tethered to the helicase (in green) by the fork protection complex (in blue). In the presence of inhibitors of replicative DNA polymerases or DNA distorting lesions (red stars), polymerases stall while the helicase continues to unwind DNA producing an excess of unreplicated ssDNA that constitutes the primary substrate for checkpoint signaling. (**B**) Other DNA lesions, such as interstrand crosslinks produced by mitomycin C, or specialized DNA and/or chromatin structures, and perhaps proteins bound to DSBs, primary halt the progression of the helicase and stall DNA polymerases since ssDNA is not immediately produced. This can be produced at a later time as a result of resection of a DSB.

The most common ATR activation signal involves all processes interfering with DNA replication fork progression, a situation also known as replication stress. Replication stress can be induced by DNA damage or by inhibition of replicative DNA polymerases activity, either using hydroxyurea (HU) that deplete the cellular pool of deoxyribonucleotides or using aphidicolin, a competitive inhibitor of DNA polymerases. Certain types of DNA damage can induce replication stress since they alter the three dimensional structure of the DNA helix (such as UV, cisplatin, camptothecin amongst others). As a consequence, DNA polymerase activity is reduced but not that of the helicase, since it can translocate through such lesions. In these situations, a functional uncoupling between the enzymatic activities of both DNA polymerases and helicase occurs (Figure 3). The result of this process is the production of excess ssDNA, a process commonly known as replication fork uncoupling [21,89]. Replication stress that produces an excess of ssDNA results in an increased level of the major ssDNA binding protein RPA bound onto chromatin. Curiously, in *S. cerevisiae*, cells treated with HU only generate a very limited amount of ssDNA (up to 200 nucleotides per replication fork) [90]. In contrast in metazoans, the length of ssDNA at stalled replication fork is much higher and can reach several kilobases [21,91]. The functional and molecular bases of this difference are to date unknown. Other types of DNA damage, such as interstrand cross-links, physically inhibit the helicase and by consequence also block the activity of DNA polymerases, since ssDNA formation is reduced, resulting in replication fork stall without immediate production of excess ssDNA [75]. However, a limited amount of ssDNA can be generated at forks stalled by DSBs following their processing (resection) by the coordinated action of nucleases, DNA helicases and associated proteins ([92] for review). Resection is initiated by the Mre11, Rad50, Nbs1 complex (MRN) and the Sae2/Ctlp/Ctp1 endonucleases in a 5'–3' direction in a process that requires CDK activity. More extensive resection occurs upon recruitment of the endonuclease EXO1 (MUS81-EME1, in fission yeast), the RecQ/Sgs1/BLM helicase and the DNA2 endonuclease. DSB resection generates RPA-coated ssDNA that in turns further stimulates this process. CtIP has been shown to be also a necessary actor in the DSB resection in cooperating with MRN complex for full ATR activation [93]. Other findings have shed light on a distinct mechanism of how an ATR-dependent DNA damage response is mediated by Apurinic/apyrimidinic endonuclease 2 (APE2) in the oxidative stress response. APE2 promote recruitment of ATR, ATRIP, and Rad9 to damage sites and thus participates in oxidative stress-induced Chk1 phosphorylation [94].

Double strand breaks are thought to induce replication fork collapse since the replisome would just unload from the DNA. Although this is the case *in vitro* on naked DNA, double strand breaks *in vivo* are likely bound by specific proteins, such as ATM and the MRN complex for instance, which may in principle constitute a barrier to the progression of the helicase, thus resulting in stalling of the replication fork without production of excess ssDNA. Interestingly, a physical direct interaction between ATM and PCNA has been recently reported in mammalian cells [95].

### 5.3. Basic Model for ATR Activation at Stalled Replication Forks

The current model for ATR activation assumes that ATR is recruited onto ssDNA via an interaction with its cofactor ATRIP (ATR-Interacting Protein) and RPA [96,97]. ATR associates with ATRIP in a stable stoichiometric complex independently of DNA damage. ATRIP binds directly to RPA-coated ssDNA, and is thought to target ATR at sites of replicative stress or DNA damage [98,99]. After induction of damage, ATR and ATRIP are located in DNA damage nuclear foci [99] and, in human cells, formation of these foci is inhibited when the expression of RPA is downregulated by RNA interference [97,100]. In addition, down regulation of RPA also result in inhibition of checkpoint activation. These findings have led to the proposal that nucleation of RPA onto ssDNA generated at stalled forks is a key determinant of checkpoint activation. However, more recent data in *Xenopus* egg extracts show that recruitment of excess RPA at stalled forks is dispensable for S-phase checkpoint activation, while generation of ssDNA is absolutely required [101]. These results are consistent with previous observations in mammalian cells showing that RPA is not quantitatively required for checkpoint activation [102] and that, in yeast, an RPA mutant that cannot interact with the checkpoint clamp 9-1-1 complex (see below) is still checkpoint proficient [103]. Finally, an ATRIP mutant that cannot interact with RPA was also shown to normally activate the checkpoint in mammalian cells [98]. In addition to its participation in checkpoint activation, RPA is primarily required for the establishment of replication forks (see Section 2.3). Thus, without RPA, replication forks cannot be made and therefore ssDNA is not stabilized. Hence, it is very likely that removal of RPA from the cell mainly inhibits checkpoint activation indirectly—by inhibiting DNA synthesis (see below)—an issue that was not previously fully taken into account [104]. Interestingly, some of us have shown that reduced levels of RPA induce formation of excess ssDNA and ATR activation, and that in S-phase, ssDNA is not processed by exonucleolytic activities [101]. This is not surprising knowing that many other ssDNA binding proteins exist in the cell that may protect ssDNA from cleavage [105]. It is thus unlikely that in the presence of low levels of RPA, ssDNA is present under naked, unprotected form in the cell. However, this substrate may be unstable in mitosis, when cells attempt to segregate chromosomes with underreplicated DNA, since it can fuel recombination-based mechanisms, as previously proposed [106]. Based on these observations, some of us predicted that hypomorphic mutations in RPA or other replication proteins might be a source of replication stress that can trigger genomic instability. In line with this possibility, a hypomorphic mutation in RPA was previously shown to induce genetic instability and fuel lymphoma in mice [106], and recently, genomic instability has been observed also in human cells in which RPA is limiting [107]. In this report, ATR is also suggested to be important to limit formation of ssDNA upon replication stress, by inhibiting firing of dormant origins (see Section 5.6).

Activation of ATR kinase activity requires recruitment of important ATR activators, the checkpoint clamp Rad9-Hus1-Rad1 (9-1-1 complex) and TopBP1 [108]. The heterotrimeric 9-1-1 complex is loaded at stalled replication forks in a similar manner to PCNA, which is loaded by RF-C, except that loading of this checkpoint clamp complex involves an RF-C-like complex in which the largest subunit of RF-C (RFC1) is replaced by Rad17 (RFC^Rad17^) [109,110]. It has been demonstrated that binding of the 9-1-1 complex requires the presence of a 5'-end at the junction between double and ssDNA [111,112]. Interestingly, although the recruitment of the 9-1-1 complex and ATR onto chromatin are independent and both necessary for full ATR activation [111], an *in vitro* study in *S. cerevisiae* suggests a direct interaction between the *C*-terminal part of Ddc1 with Mec1 (respectively RAD9 and ATR homologues in *S. cerevisiae*, see Table 1) [113].

TopBP1 is a large, multi-functional protein, playing an essential role in both DNA synthesis and checkpoint activation [114,115]. These functions of TopBP1 come under separable domains: the N-terminal domain is necessary for activation of DNA synthesis, while the C-terminal domain is required for checkpoint activation [116,117]. Although TopBP1 function appears not to be essential during ongoing DNA synthesis, this protein is found associated with unperturbed replication forks [29,118] and may stimulate ATR activity by local specific protein-protein interactions during replication fork progression to coordinate the temporal order of origins firing. Several additional interactions have been described for TopBP1 in replication checkpoint. Interaction between the phosphorylated tail of Rad9 (on serine 387) with BRCT repeats I and II of TopBP1 [119,120,121] and another interaction between TopBP1 with ATR-ATRIP through a specific domain known as ATR-Activation Domain or AAD [122,123] localize TopoBP1 for ATR activation. Recruitment of high levels of 9-1-1 on chromatin require TopBP1 [124] and stabilization of TopBP1 at stalled forks has been shown to be also dependent upon the DNA helicase FANCJ/BACH1 [125]. Finally, interaction between TopBP1 and the MDC1 (Mediator of the checkpoint 1) protein has been shown to be important for Chk1 phosphorylation after replication stress [126]. Recently, the MRN complex has also been implicated in ATR activation through stabilization of TopBP1 [127] and this function appears to depend upon its catalytic activity [128]. Altogether, these findings highlight a complex structural network of interactions mediated by TopBP1 whose function in the context of the stalled replication forks will be very interesting to elucidate in the future.

**Figure 4:**
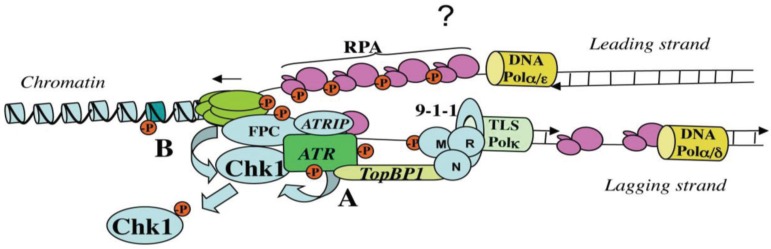
Speculative model for replication checkpoint activation. Replication fork uncoupling generates excess ssDNA (see Figure 3) that is coated by RPA. On the lagging strand DNA polymerase, α and δ replicative polymerases catalyze synthesis of short replication intermediates (priming reaction). It is not yet clear whether these intermediates are also generated on the leading strand, and if this is the case, which is the DNA polymerase implicated (question mark). A strong candidate is the recently identified Primpol enzyme [135,136,137,138,139]. TLS DNA Pol κ may be implicated in recruitment of the 9-1-1 complex by stabilization and/or synthesis of short replication intermediates in proximity of the ATR-ATRIP complex thus facilitating interaction via TopBP1. The MRN complex may facilitate the interaction between TopBP1 and ATR by an as yet unknown mechanism. Once activated, ATR may autophosphorylates and phosphorylate chromatin-bound as well as soluble substrate (-P). CHK1 is phosphorylated by ATR (**A**), and phosphorylation is potentiated *via* interaction with Mrc1/Claspin within the FPC (**B**), although this can also occur in the soluble fraction of the nucleus. The green histone on the chromatin represents H2AX.

Previous observations have proposed a role for DNA synthesis in ATR activation [129,130]. DNA primase/polymerase activities were shown to be required for ATR activation at stalled replication forks. Since this enzyme is absolutely required to initiate DNA synthesis, these data can be interpreted as an indirect role for DNA polymerase α in stimulating replication forks formation. This is well illustrated by the observation that the checkpoint is inactive when initiation of DNA synthesis is inhibited by Geminin [101,129]. However, a recent study has addressed the requirement of DNA synthesis in DNA replication checkpoint [131] and convincingly showed that continued synthesis and elongation of new DNA primers on ssDNA templates occurs at replication forks stalled with aphidicolin. Since synthesis of these replication intermediates is essential for binding of the 9-1-1 complex, these findings highlight an additional key step in ATR signaling. Formation of ssDNA at arrested forks may be then important to generate replication intermediates that stimulate recruitment of the 9-1-1 complex catalyzed by the RFC^Rad17^ complex. Once loaded, the 9-1-1 checkpoint clamp is tethered to the ATR-ATRIP complex via the TopBP1 C-terminus, thus leading to ATR activation (Figure 4). Nonetheless, it remains unclear what the precise molecular substrates are that allow ATR activation. Recent data suggest that autophosphorylation of ATR is important for its kinase activity. Autophosphorylation appears to be dependent on RPA, 9-1-1, and not TopBP1 [132]. A similar model based on autophosphorylation was previously proposed for ATM activation [133], suggesting a conserved mechanism of activation within the PI3K family. ATM activation has also been shown to require generation of small DNA fragments [134]. Short replication intermediates synthesized onto ssDNA generated at stalled forks would be the equivalent substrate that stimulates ATR activation (see below). Interestingly, ATR can also be activated following processing of a DSB by end-resection (see Section 5.2), but the role of small DNA fragments generated in this process remains to be determined. Alternatively, ATR may be activated in a different manner in specific situations.

### 5.4. Role of Replication Fork Uncoupling and RPA Nucleation onto ssDNA DNA Replication Checkpoint Activation

High levels of RPA and DNA polymerases have been observed in *Xenopus* at forks stalled with aphidicolin or UV irradiation [21,129]. This was thought to represent a checkpoint-specific recruitment of RPA [104]. However, more recent data strongly suggest that this may not be the case, since nucleation of neither ATRIP [98], nor DNA polymerase α [131] or RPA [101] appear to be required for checkpoint activation at arrested forks. Moreover, it has been shown that in *Xenopus* egg extracts, when initiation of DNA synthesis is poisoned by high concentration of aphidicolin, RPA nucleates at unwound forks, but the 9-1-1 clamp does not load and checkpoint activation does not occur [112]. These observations are entirely consistent with the mode of checkpoint activation of the yeast *S. cerevisiae* that occurs without detectable RPA nucleation at stalled forks [90]. Finally, checkpoint activation occurs spontaneously in *Xenopus* extracts containing a limited amount of RPA that do not support its hyperloading at replication forks arrested with aphidicolin [101]. In this situation, DNA synthesis is somehow slow and nuclei accumulate high level of ssDNA, suggesting spontaneous replication fork uncoupling. Chronic checkpoint activation has also been observed in yeast Ctf-4 mutants that are defective in the coupling between DNA polymerases and MCM2-7 helicase [140], as well as in mutants of the Mrc1 gene [141], a subunit of the Fork Protection Complex (FPC, see Section 5.5). Taken together, these observations strongly suggest that the basic feature of replication checkpoint activation, conserved from yeast to vertebrate, is the uncoupling of DNA synthesis regardless of the length of the ssDNA produced. Hence primed ssDNA generated at stalled forks may be the natural substrate that facilitates formation of the ATR-ATRIP-9-1-1-TopBP1 complex leading to ATR full activation (Figure 4). This process constitutes a fine-tuning mechanism that efficiently senses progression of S-phase leading to various degrees of checkpoint activation, depending on the extent of replication fork uncoupling. Furthermore, it does not require a specific loading of DNA polymerase α, TopBP1 or RPA onto chromatin, since these proteins are already bound at the replication forks during a normal S-phase, and recycle onto ssDNA after completion of their catalytic activity [34,101,131]. ATR itself binds to chromatin in a replication-dependent manner in the absence of replication stress [142]. This model is also consistent with observations in both yeast and mammalian cells that colocalization of checkpoint sensors is a minimal sufficient requirement for checkpoint activation even in the absence of external damage [143,144,145,146]. The question remains of whether primed ssDNA is also made on the leading strand at arrested forks, and if this is the case, which of the DNA polymerase fulfills this task. An elegant study in *E. coli* has previously shown that the DNA damage tolerance system that involves translesion DNA polymerases is required to resume DNA synthesis at replication forks arrested by a DNA lesion generated by the carcinogen AAF [77], which is consistent with the observation that primer DNA synthesis continues at arrested forks in *Xenopus* egg extracts [131]. Recent work has provided evidence that one of these enzymes, TLS Pol κ, is involved in DNA synthesis at arrested forks in eukaryotic cells, in particular when forks are blocked with aphidicolin [147], since translesion DNA polymerases are not sensitive to this drug. TLS pol κ was shown to facilitate binding of the 9-1-1 complex at arrested forks, probably by direct physical interaction and, therefore, contributing to full Chk1 phosphorylation.

### 5.5. Cellular Responses to ATR Activation

#### Mediators and Transducers of the S-Phase Checkpoint

ATR activated at arrested forks phosphorylates various substrates to coordinate cell cycle arrest, maintenance of replication fork stability, origin firing and eventually fork restart. ATR phosphorylates soluble substrates as well as chromatin bound substrates (Figure 2 and Figure 4). Probably the best ATR characterized chromatin-bound substrate is the histone variant H2AX, known as γH2AX [148]. Phosphorylation of H2AX occurs by ATR after replication stress induced by aphidicolin, hydroxyurea or DNA distorting lesions, such as those induced by UV damage or cisplatin. An important function of H2AX phosphorylation at arrested forks appears to allow recruitment of the checkpoint mediator MDC1. Once recruited, MDC1 would act as a scaffold to recruit additional activated ATR molecules and propagate H2AX phosphorylation over a long distance, producing the characteristic pan nuclear staining observed by immunofluorescence [126]. Amongst other chromatin-bound ATR substrates there are MCM2-7 proteins [149], although the specific role of their phosphorylations remains unclear [150]. The RPA2 subunit of the RPA complex has also been shown to be phosphorylated by ATR upon replication stress [151,152,153]. Recent work has shown that phosphorylation mutants of RPA2 do not interfere with RPA binding to ssDNA and that this post-translational modification is not required for activation of the replication checkpoint in both *Xenopus* and human *in vitro* systems [154,155]. Another study suggests that this modification may be required to resume DNA synthesis facilitating replication fork restart [156]. Other replication substrates include subunits of the ORC complex, DNA polymerase ε, the RF-C complex and RPA1 [79]. In the future, it will be interesting to determine the role of these specific phosphorylations upon the structure/function of the replisome. Mrc1/Claspin can also be considered as a mediator of the replication checkpoint since mediates the autophosphorylation loop of the CHK1 protein kinase ([157] and see Section 5.6) by direct interaction [158]. Mrc1/Claspin is also phosphorylated by ATR and this phosphorylation appears to be important in mediating hyperphosphorylation of CHK1 [158,159,160]. Claspin/Mrc1 associates with two other proteins—Timeless/Tof1/Swi1 and Tipin/Swi3/Csm3—forming the Fork Protection Complex (FPC) that supports CHK1 phosphorylation [161]. FPC is bound to chromatin also during the unperturbed cell cycle and mutation in Mrc1 in yeast induce chronic replication fork uncoupling in the absence of external damage [136], suggesting an important role of FPC in coordinating helicase and DNA polymerases activities. Consistent with this conclusion, subunits of the FPC have been found to interact with MCM2-7 proteins [162] and RPA [163]. Interestingly it has been shown that Claspin binding to chromatin is not essential for CHK1 phosphorylation in *Xenopus* egg extracts [164], showing that both chromatin-bound and nuclear-soluble forms of the Claspin-CHK1 complex can exist and be functional.

### 5.6. Transducers

Amongst soluble ATR targets there is CHK1, a well-known serine-threonine kinase. Once phosphorylated, CHK1 prevents S-phase progression by targeting key cell cycle regulators. More precisely, ATR phosphorylates CHK1 on two serine residues, S^317^ and S^345^ in human cells [165,166] and these phosphorylations are enhanced by the mediator protein Mrc1/Claspin. One important aspect of ATR activation is the regulation of late origins firing. Previous work has strongly suggested that the main function of ATR is to reduce CDK2 activity so as to inhibit further firing of licensed replication origins [167,168]. This occurs through CHK1-mediated phosphorylation of the CDC25A phosphatase targeting it for proteasomal degradation. Reduction of CDC25A activity in turn inactivates CDK2/Cyclin E through inhibition of CDK2 tyrosine 15 dephosphorylation. In this situation, the CDK-dependent step of pre-IC complex assembly cannot occur and replication origins will not fire. Interestingly, the identification of replication origins that fire in such a context has been described. These are the so-called dormant origins [169], and may represent origins that have the potential to fire but are actively repressed by nearby activated origins. The molecular mechanism underlying activation of these origins is yet to be discovered, although ATR is implicated [107]. Importantly, failure to activate dormant origins results in DNA damage [170]. Another important aspect of ATR activation has been for long considered the stabilization of the replisome so as to avoid collapse of arrested or paused replication forks. Very recent data have now challenged this model showing that forks are stable during a replication forks arrest in checkpoint mutants and suggest that phosphorylation of replisome components may be involved in maintenance of intact arrested replication forks ([171] and references therein).

### 5.7. Effector Proteins

The phosphorylated form of CHK1 phosphorylates many targets. Recently, the cellular targets of this kinase have been identified and found to be soluble as well as chromatin-bound [172]. The function of CHK1 phosphorylation of many of these substrates is still unknown. However, molecular targets that modulate CDK activity and in turn slow down progression of the cell cycle (effector proteins) have been identified. One of the most extensively studied direct CHK1 targets is the master cell cycle phosphatase CDC25A. CHK1 phosphorylates CDC25A on Serine 76, which promotes its ubiquitin-mediated destruction in order to prevent activation of CDK activity during S-phase. Although this phosphatase activates both CDK2/Cyclin E and CDK2/Cyclin A complexes during the G1 to S-phase transition [173], it also has been implicated in the G2 to M-phase transition and during S-phase upon detection of DNA damage [174]. During mitosis, CDC25A is stabilized while at mitotic exit CDC25A is targeted for proteasomal degradation by the anaphase promoting complex/cyclosome (APC/C). Upon DNA damage, multiple checkpoints can be activated aiming at reducing CDK activity and allowing DNA repair. CDC25A is a very unstable protein which abundance is regulated during progression of the cell cycle [175]. The importance of a tight regulation of CDC25A abundance is underscored by the observation that it is overexpressed in many human cancers [176] as well as in fast proliferating mouse embryonic stem cells [177].

## 6. Conclusions

The replication checkpoint is a fine-tuned mechanism that allows rapid slow down of S-phase in the presence of internal of external cues that interfere with DNA synthesis, so as to preserve genome integrity. Analysis of tumors at early stages of transformation has revealed the presence of a high degree of replication stress [178,179]. This is believed to be the result of oncogene activation but how oncogenes activation triggers replication stress is to date unclear [180]. Future work focusing on this issue would be required to give a physical identity to replication stress, for instance by identifying DNA structures that are specifically generated by replicative stress. Replication fork arrest constitutes the phenotypic manifestation of replication stress. This situation is dangerous since prolonged replication fork arrest may induce activation of DNA replication restart and/or recovery pathways, one of which is recombination. Excess ssDNA produced at arrested forks can also be deleterious since this substrate could in principle be processed by exonucleolytic cleavage. Though, RPA binding onto ssDNA has been important to prevent its degradation. However, in *Xenopus* egg extracts, a large amount of ssDNA can be generated after replicative stress, and it is observed that they are not targeted by exonucleolytic cleavage during S-phase [101] nor replication fork collapse (our unpublished observations). It could be possible that other ssDNA binding protein may protect ssDNA from exonucleolytic degradation or cleavage in the absence of RPA and/or that breakage may occur during mitosis. In the future, it would be interesting to identify the proteomic composition of ssDNA and determine the role of ssDNA binding factors in ssDNA metabolism and/or stability.

Although the ATR pathway has been extensively investigated in the past decade, there are still many unresolved questions that are yet be addressed. Why so many proteins are needed to promote full ATR activation? Is ATR binding to a specific DNA structure? What is the role of ATR autophosphorylation? How different activities are coordinated at stalled replication forks? Moreover, what is the defined function of the 9-1-1 complex bound to stalled forks? Are there any replication intermediates that are generated on the leading strand at stalled forks? What is the contribution of these structures to DNA replication checkpoint activation? Are there any other translesion DNA polymerases implicated in DNA synthesis at stalled replication forks? These unresolved questions and many more related to the mechanism of ATR activation will be the challenging task of future investigations.
